# Multifunctional Role of ATM/Tel1 Kinase in Genome Stability: From the DNA Damage Response to Telomere Maintenance

**DOI:** 10.1155/2014/787404

**Published:** 2014-08-28

**Authors:** Enea Gino Di Domenico, Elena Romano, Paola Del Porto, Fiorentina Ascenzioni

**Affiliations:** ^1^Pasteur Institute-Cenci Bolognetti Foundation, Department of Biology and Biotechnology “Charles Darwin”, Sapienza University of Rome, 00185 Rome, Italy; ^2^Department of Biology and Biotechnology “Charles Darwin”, Sapienza University of Rome, 00185 Rome, Italy

## Abstract

The mammalian protein kinase ataxia telangiectasia mutated (ATM) is a key regulator of the DNA double-strand-break response and belongs to the evolutionary conserved phosphatidylinositol-3-kinase-related protein kinases. ATM deficiency causes ataxia telangiectasia (AT), a genetic disorder that is characterized by premature aging, cerebellar neuropathy, immunodeficiency, and predisposition to cancer. AT cells show defects in the DNA damage-response pathway, cell-cycle control, and telomere maintenance and length regulation. Likewise, in *Saccharomyces cerevisiae*, haploid strains defective in the *TEL1* gene, the ATM ortholog, show chromosomal aberrations and short telomeres. In this review, we outline the complex role of ATM/Tel1 in maintaining genomic stability through its control of numerous aspects of cellular survival. In particular, we describe how ATM/Tel1 participates in the signal transduction pathways elicited by DNA damage and in telomere homeostasis and its importance as a barrier to cancer development.

## 1. Introduction

Ataxia telangiectasia mutated (ATM) is a protein kinase member of the evolutionary conserved phosphatidylinositol-3-kinase- (PI3 K-) related kinase (PIKK) family [1, 2]. The PIKK family members are serine/threonine protein kinases (270–450 kDa) characterized by N-terminal HEAT repeat domains and C-terminal kinase domains [3]. ATM is a relatively large protein with a molecular weight of 350 kDa and consisting of 3056 amino acids [4]. The C-terminus kinase domain of ATM is flanked by two regions called FAT (FRAP, ATM, and TRRAP) and FATC (FAT C-terminus), which participate in the regulation of the kinase activity [5]. The rest of the protein contains HEAT repeats at the N-terminus that mediates protein and DNA interactions [6].

Patients with ATM deficiency are affected by the human autosomal recessive disorder ataxia telangiectasia (AT), a rare neurodegenerative disease that causes multiple stress symptoms, including cerebellar degeneration, increased incidence of cancer, growth retardation, immune deficiencies, and premature aging [7]. AT was first described in 1957, as a distinct disease that can occur early in childhood, with incidence varying from 1 out of 40,000 to 1 out of 100,000 new births and a carrier frequency that approximates 1% [8]. Several hundred ATM mutations have been identified in AT patients, most of which are heterozygous and inherit different AT mutations from each parent [9]. About 85% are null mutations that result in the production of truncated forms of the protein and complete inactivation of the gene function [10], while less than 15% are classified as missense mutations [11]. At the cellular level, ATM deficiency correlates with hypersensitivity to DNA-damaging agents. Accordingly, after DNA double-strand breaks (DSBs), ATM/Tel1 and ATR/Mec1 (ataxia telangiectasia Rad-3-related/yeast ortholog Mec1), which are categorized as DNA-damage checkpoints, become active and start the signal transduction pathways that block the cell cycle and repair the DNA damage or eventually activate the cell death program. Thus, as a consequence of dysfunctional ATM signaling, different effects have been reported, such as reduced phosphorylation levels of DNA damage response (DDR) targets [12], failure to arrest the cell cycle, and reduced efficiency of DNA damage repair [13–16]. Additionally, telomere associations are frequently observed in cells derived from individuals with AT [17, 18], and cells expressing dominant negative ATM variants show accelerated telomere shortening [19, 20].

*TEL1* (telomere maintenance 1), the* Saccharomyces cerevisiae* ortholog of human ATM, was identified in a screen for genes that affect telomere length [21].* TEL1* encodes a very large (322 kDa) protein that shares 45% amino-acid identity in the kinase domain and 21% amino-acid identity in the rest of the protein with the human ATM gene [22, 23]. Similar to* ATM* and together with* MEC1*,* TEL1* is a key regulator of the checkpoint response to DSBs. Additionally, yeast cells lacking Tel1 have short but stable telomeres that consist of about 50 bp telomeric repeats, which corresponds to a sevenfold reduction to that reported in wild-type strains [21]. According to the prevailing model, the major role of Tel1 is the promotion of preferential lengthening of short telomeres. However a number of experimental observations do not fit with this theory, which suggests that Tel1 has a more complex role in telomere maintenance [24–26].

Despite the differences between humans and budding yeast, what emerges is that ATM/Tel1 is a key element in the detection and signaling of intrachromosomal DSBs and in the maintenance of telomere metabolism. In this review, we discuss the dual role of ATM/Tel1 in the sophisticated surveillance mechanisms at DSBs and in telomere regulation, to highlight the overall importance of its dual nature in genome stability and long-term cell survival.

## 2. Activation of ATM/Tel1 in Response to DNA Damage

The DDR comprises different surveillance mechanisms that guarantee genome stability and cell survival in response to DNA damage. The generation of simultaneous breakage of the two complementary DNA strands prompts activation of DSB repair mechanisms, delay or arrest of cell-cycle progression, and eventually programmed cell death [27]. All eukaryotes, from human to yeast, have evolved conserved mechanisms to protect the genome from DSBs, which mainly relies on the PIKK members ATM/Tel1 and ATR/Mec1. In vertebrates there is a third member of the PIKK family called DNA-dependent protein kinase catalytic subunit (DNA-PKcs) that has a direct role in DNA DSB repair and DNA damage signalling. However, DNA-PKcs results are to be dispensable in most eukaryotes and it has no homologue in* S. cerevisiae* or* Saccharomyces pombe* [28]. In vertebrates DNA-PKcs functions together with the Ku heterodimer as a DNA end-bridging factor and in association with the MRN complex tether the DNA ends of DSB together [29–31]. In* S. cerevisiae*, the MRX complex appears to have the bridging activity role alone, which obviates in this way the role of DNA-PKcs [32, 33].

Erroneously, Mec1 was long considered the primary sensor of DNA damage, as its loss results in severe sensitivity to DNA-damaging agents, while the absence of Tel1 does not significantly affect cell survival under these conditions [22, 23, 34]. However,* mec1, tel1* double mutants reveal an increased sensitivity to genotoxic agents with respect to the single mutants [23, 35–37], which suggests that Tel1 has a key role in the DSBs response and acts on a different epistasis group with respect to Mec1. Misinterpretation of data obtained with* mec1* or* tel1* mutants and analysis of sensitivity to genotoxic agents can be explained by the ability of yeast* tel1* mutants to rapidly convert DNA damage into substrates that preferentially activate the Mec1 kinase. Indeed, ATM/Tel1 and ATR/Mec1 respond to specific DNA damage. While ATM/Tel1 is activated by blunt end or minimally resected DSBs, DNA lesions that lead to extended resection and generation of long replication protein A- (RPA-) coated single-strand (ss) DNA activate ATR/Mec1. This DNA damage specificity appears to be conserved in human and yeast.

Two major pathways are involved in the repair of the DSBs: nonhomologous end-joining (NHEJ) and homologous recombination (HR). NHEJ is active throughout the cell cycle and relegates broken ends without the need of extensive processing [38]. NHEJ is efficient in the repair of the damage, but it can cause mutations at the joining sites. On the contrary HR, is more accurate and requires the presence of long and undamaged 3′-ssDNA to repair the broken ends, typically the sister chromatid. Consequently, HR is limited to S/G2 phase [39]. ATM/Tel1 and ATR/Mec1 regulate the DNA damage signalling response. In particular ATM is activated by DSBs, while ATR is activated at single-strand regions of DNA via a process that involves ATRIP, RPA, and the presence of long stretch of ssDNA. In both human cells and* S. cerevisiae*, ATM/Tel1 is recruited at blunt or minimally resected DNA ends by the MRN/MRX complex [37, 40]; therefore, cells that experience DNA damage in G1 are prevented from entering S-phase by the G1/S checkpoint signalling cascade that is dependent on the activity of ATM [41]. In S phase, ATR can be activated by replication fork stalling/collapse [42]. In G2 phase, DSBs can be resected via an ATM-dependent process generating ssDNA that can activate ATR following RPA association [43]. RPA complex binds to the ssDNA tails and recruits the ATR/Mec1 checkpoint kinase. Therefore, the resection process during DSB represents a central event not only to drive the DSB repair by NHEJ or HR, but also to trigger the specific ATM/Tel1 or ATR/Mec1-checkpoint response.

**Figure 1:**
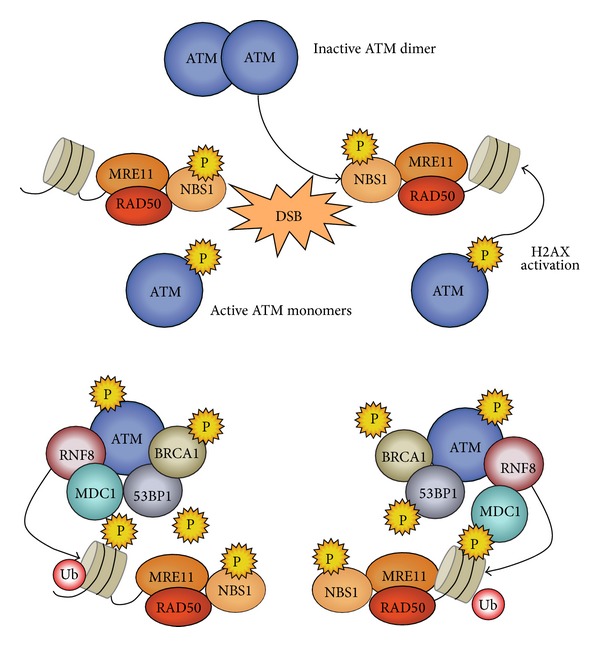
Description of the relevant proteins recruited to DNA double-strand break. In undamaged cells, ATM is an inactive multimer. After DSBs, ATM is recruited to the site of damage by the MRN complex, triggering its autophosphorylation, monomerization, and subsequent activation. Adjacent to the site of damage, the first target of ATM is the histone H2AX, followed by the phosphorylation of MDC1 and the recruitment of the ubiquitin ligase RNF8. RNF8 binding causes H2AX ubiquitylation, facilitating the association of BRCA1 and, ultimately 53BP1, that is required for ATM retention at the site of damage.

The first evidence of the primary role of ATM in DDR came from the study of AT patients. Since 1967, it has been reported that AT patients show an unexpected hypersensitivity to ionizing radiation [44], and cells from these patients exhibit pronounced sensitivity to DNA-damaging agents, failure of checkpoint signaling, imperfect DSB repair, or variable defects in apoptosis [14, 16, 45]. In cells, under physiological conditions, ATM is present as inactive dimers or higher-order multimer [46]. After DNA damage, ATM is converted into partially active monomers (Figure 1), which requires the autophosphorylation on S1981 and its interaction with MRN at the DSBs [46–49]. Despite the fact that ATM autophosphorylation of S1981 represents a marker of activation the real contribution of S1981 phosphorylation in ATM activation remains unclear.* In vitro* experiments suggest that ATM monomerization by MRN does not require ATM S1981 autophosphorylation [50]. Mouse models bearing an ATM-S1987A mutation (equivalent of the human S1981A), expressed on an Atm^−/−^ background, or S1987A mutation with two additional autophosphorylation site mutations (corresponding to human S367A and S1893A) showed no defects in ATM activation [51–53].

Other phosphorylation sites, identified by mass spectrometry in cells exposed to ionizing radiations (S367, S1893, and S2996) [54], appear to be involved in ATM activation, as suggested by the finding that the S1981 mutant (S1981A) can still form monomers [50]. In postmitotic neurons, ATM is phosphorylated at S794 by cyclin-dependent kinase 5 (CDK5), followed by the autophosphorylation of S1981 [55]. Acetylation of ATM by the acetyltransferase Tip60 is required for complete activation of ATM [56, 57]. Overall, the precise mechanisms involved in ATM activation remain to be fully elucidated. It has been observed that dysfunction in any components of MRN complex prevents ATM activation [40, 48–50] whereas ATM recruitment to DSBs relies on the interaction with the NBS1 [40, 46, 48, 50], and its retention appears to be dependent on Mre11 [58]. ATM activation is inhibited in the presence of DSBs induced by H_2_O_2_ as oxidation blocks the ability of MRN to bind to DNA. Nevertheless, the addition of H_2_O_2_ to purified dimeric ATM* in vitro* stimulates its activity on a p53 substrate even in the absence of MRN. These results suggest that ATM can be activated directly by oxidative stress through an MRN/DSB-independent mechanism [59, 60].

The NBS1 component of the MRN complex contains a PIKK carboxyl-terminal motif that interacts with ATM, thus promoting recruitment and activation of ATM, which in turn activates the signaling cascade involved in the DNA repair [48].* In vitro* experiments have shown that ATM activation is achieved when Nbs1 forms a complex with Mre11 and Rad50 and not by itself [50]. Together with the findings that, in the absence of MRN, ATM does not respond to DSBs, this suggests that the MRN complex acts as a central coordinator of DDR. Indeed, MRN physically localizes to the DSB site immediately after the damage and promotes end resection [61–63]. One of the first events following DNA breakage is end resection, which leads to ssDNA generation. MRE11 together with CtIP carries out limited resection of DSBs, which is subsequently extended by the activity of nucleases and helicases such as EXO1, BLM, and DNA2 [64]. This occurs via two pathways: in one, the Bloom helicase (BLM) and the ssDNA helicase/nuclease DNA2 physically interact and promote 5′-3′ DNA resection, a process that is stimulated by RPA. In a second pathway, BLM, MRN, and RPA promote recruitment of the exonuclease EXO1 to the broken ends and stimulate resection [65].

When ATM is activated by MRN, its phosphorylation level oscillates during DSBs repair, due to the activity of phosphatases [66]. Studies carried out with human cell lines have revealed that the protein phosphatase 2A (PP2A) can constitutively dephosphorylate ATM, thus acting as a negative regulator of the DSB repair process [67], although it has been shown that inhibition of PP2A activity can cause defective DNA damage repair [68–70]. One possible explanation for this discrepancy relies on the presence of several distinct PP2As, which directly dephosphorylates ATM at various sites (S367, S1893, and 1981), thus modulating its retention at DSB sites [71]. Other phosphatases are also involved in ATM regulation, including protein phosphatase 5 (PP5), the interaction which with ATM increases after ionizing radiations exposure [72, 73], and the wild-type p53-induced phosphatase 1 (WIP1), which specifically dephosphorylates the ATM S1981 residue [74].

Some experimental evidence has suggested that efficient DSB repair also requires chromatin remodeling, which is triggered by ATM-dependent phosphorylation on S139 of the histone H2A variant (*γ*-H2AX). This type of histone modification spreads over about 2 Mb surrounding a break [75, 76]. Additionally, chromatin relaxation in the area surrounding DNA damage [77] potentiates the ATM signaling and radioresistance [78], as demonstrated by using histone deacetylase inhibitors and chromatin-modifying agents, such as chloroquine or osmotic shock [46]. Moreover, it has been suggested that, by regulation of the level of acetylation of Lys 14 of histone H3 (H3 K14) before and after DSBs, the nucleosome-binding protein HMGN1 optimizes activation of ATM [79]. On the other hand, DNA repair in heterochromatic regions is facilitated by ATM-mediated transient chromatin relaxation, through phosphorylation of KRAB-associated protein 1 (KAP1) at residue S824 [80, 81]. Accordingly, depletion of KAP1 rescues the radiosensitivity of cells treated with ATM inhibitors [82].

The first model that described the role of ATM in DDR was proposed on the basis of experimental data obtained in* S. cerevisiae* and demonstrated that Xrs2 (homolog of NBS1) interacts with Tel1 through its C-terminal region, thus providing the molecular basis of MRX-dependent recruitment of Tel1 to DSBs [40]. In contrast with ATM, activation of Tel1 has not been extensively studied as it was long considered to be redundant with Mec1. Indeed, Tel1 mutants do not exhibit increased sensitivity to genotoxic agents and Tel1 phosphorylates some of the Mec1 substrates only in the absence of Mec1 [35, 37] and it can activate the DDR independently of Mec1 only in the presence of multiple DNA breaks [37]. In S phase, when Tel1 is deleted and, in the presence of a Dna2 mutant, the phosphorylation of Rad53 and Mrc1 is partially reduced [83], the apparent minor role of Tel1 in the DDR may be somewhat explained by the ability of* S. cerevisiae* to efficiently convert DSB ends into ssDNA that activate Mec1 kinase activity.

In* S. cerevisiae*, similar to mammalian cells, ssDNA production at DSBs results from a two-phase process. In the early step of resection, the MRX complex and Sae2 (ortholog of human CtIP) endonuclease create short 3′ overhangs [84, 85]. Subsequently, two alternative pathways extend the ssDNA region: one depends on the Sgs1 helicase (ortholog of human BLM) and the conserved helicase/nuclease Dna2, while the other relies on the Exo1 nuclease [85–88].

Experimental evidence has also suggested that Sae2 is directly implicated in the activation of Tel1-mediated checkpoint signaling [89, 90]. Indeed, in cells lacking Sae2 and in the presence the genotoxic agents such as methyl methane sulfonate, Tel1-mediated Rad53 activation is potentiated, and this process requires MRX activity [90]. Additionally, Sae2 deletion stimulates the Tel1-dependent checkpoint activation in response to DSBs, by delay of MRX delocalization from damaged sites [89]. This suggests that unprocessed DNA damage accumulates in* sae*2 mutants, and when the resection cannot proceed, MRX remains stably associated to the site of damage, and Xrs2 subunit stimulates Tel1 activation, which in turn recruits Rad9 and initiates the downstream checkpoint kinase cascade [40, 90, 91] (Table 1).

Globally, these data suggest a unified model of ATM/Tel1 activation where the MRN/MRX complex is the sensor of DSBs and initiates processing of the broken ends, which in turn regulates the recruitment of the ATM/Tel1 checkpoint kinase through binding with the NBS1/Xrs2 subunit, which leads to activation of the specific downstream targets [48, 92, 93].

## 3. ATM-Tel1 Checkpoint Signaling Cascade in Response to DSBs

Proteomic studies have described nearly a thousand of potential substrates for ATM/Tel1 and ATR/Mec1, which have revealed a complex cellular response to DNA damage and cell-cycle control [94–99]. ATM/Tel1 and DNA-PKcs respond primarily to DSBs that are involved in the nonhomologous end-joining pathway of DSB repair, whereas ATR/Mec1, which shares with ATM substrates in the DSB response pathway, regulates checkpoint activation after different types of DNA damage such as UV radiations and stalled replication forks. After DSBs, MRN/MRX, ATM/Tel1, and DNA2/Sae2 promote DSB resection, to generate the initial 3′ ssDNA tails that are bound by RPA. The appearance of RPA-coated ssDNA promotes the recruitment of ATR/Mec1, which is mediated by Ddc2 (hATRIP), and which leads to localization of the Mec1-Ddc2 complex (ATR-ATRIP in human) at the site of damage. Additionally, the heterotrimeric checkpoint clamp 9-1-1 (RAD9-RAD1-HUS1 in human; Ddc1-Rad17-Mec3 in* S. cerevisiae*) is recruited independent of ATR-ATRIP/Mec1-Ddc2 [100, 101] and is required for ATR/Mec1-dependent G1 and G2 signaling, although it is dispensable for the S-phase control [102, 103]. Recruitment of the checkpoint clamp 9-1-1 appears to be also regulated by the DNA structure, as RPA restricts its loading to 5′ ssDNA/dsDNA junctions [104]. The 9-1-1 complex promotes ATR/Mec1-dependent phosphorylation of its targets, including Rad9 in yeast [105]. Once recruited, Rad9 is hyperphosphorylated and acts as a molecular adaptor that brings Rad53 into close proximity to Mec1 at sites of DNA damage, to facilitate Mec1-dependent Rad53 phosphorylation [105]. In addition it has proposed Rad9 can directly activate Rad53 increasing the local Rad53 concentration and prompting its autophosphorylation and catalytic activation [106].

Although ATR/Mec1 appears to be the major checkpoint regulator, in* S. cerevisiae* the role of Tel1 becomes evident following generation of multiple DSBs and in the absence of Mec1 [37, 90]. Accordingly, while seven HO-induced DSBs can trigger Rad53 phosphorylation and cell-cycle arrest, a single break was not sufficient to activate this response [37].

The Tel1 and Mec1 kinases are also important in the DDR and checkpoint signaling, through their modification and the remodeling activities of chromatin elements, including histones. H2A histone phosphorylation on S129 (*γ*-H2A) mediated by the Tel1 and Mec1 kinase activities is required for cell-cycle arrest in response to DNA damage during G1/S transition and to facilitate the accessibility of DNA to repair factors [107, 108].

In G1-arrested yeast cells, H2A phosphorylation depends on Tel1, which appears to be necessary and sufficient to modify the region surrounding the site of damage [109, 110]. Subsequently, as end-resection proceeds and long stretches of ssDNA accumulate, Mec1-depedent H2A phosphorylation spreads from the site of damage for about 50 kb [109, 111].

Similarly, in mammalian cells, DSBs rapidly lead to ATM- and ATR-dependent phosphorylation of histone H2AX, a variant of histone H2A, on serine 139 (*γ*H2AX). *γ*H2AX appears within several minutes after ionizing radiation and spreads along the site of damage for megabases [76, 112]. The increased density of *γ*H2AX promotes the accessibility and anchoring of other DDR proteins, such as MDC1, through its BRCT domain, which in turn promotes ATM accumulation to the sites of DNA damage [113, 114].

This sequence of events is important for the retention of ATM at DSBs (Figure 1), thus facilitating further ATM-dependent phosphorylation of H2AX and amplification of the signal [58, 76, 115]. In addition, DNA damage promotes direct ATM-dependent phosphorylation of MDC1 at the T98 site, which triggers its oligomerization and accumulation at the DSB region [116]. Once MDC1 is activated, it can recruit other proteins to DSBs, such as the RING-finger ubiquitin ligases RNF8 and RNF168 [117–120]. In particular, RNF8 promotes the *γ*H2AX histone ubiquitylation that is required for recruitment of additional DSB regulators, such as p53 binding protein 1 (53BP1) and breast cancer type 1 susceptibility protein (BRCA1), both of which are also substrates for ATM-dependent phosphorylation [120].

## 4. Cell-Cycle Checkpoints

**Figure 2:**
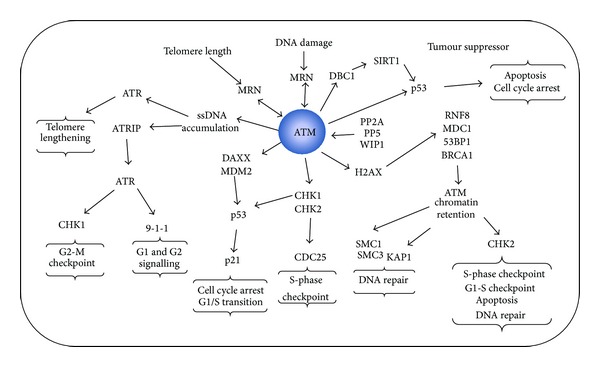
Summary of the ATM signaling network. Schematic representation of ATM signaling pathways as reported in the text.

The G1 checkpoint promotes cell-cycle arrest before the cells become irreversibly committed to the next cycle. In* S. cerevisiae*, Rad53-dependent checkpoint signaling inhibits transcription of the G1/S cyclins (Cln1, Cln2), thus inhibiting cell-cycle progression. In vertebrate, a two-step model has been proposed to explain the robust G1 checkpoint activation (Figure 2). First, ATM-dependent phosphorylation of CHK2 promotes not only CHK2 autophosphorylation, but also phosphorylation of the phosphatase CDC25A, which targets it to the proteasome. Consequently, loss of CDC25A prevents activation of the CDK2-cyclinE complex, which is required for entry into S phase. A second response depends on the tumor suppressor p53 [112].

In normal unstressed cells, p53 is a short-lived protein and its degradation is promoted by the* MDM2* (mouse double minute) gene [121, 122]. After DNA damage, ATM and CHK2 phosphorylate p53 (S15 and S20), thus reducing its ability to bind MDM2 and contributing to its stabilization [12, 41, 123, 124]. Additionally, ATM can directly phosphorylate MDM2 at S395, which leads to a reduction in MDM2 activity [125]. MDM2 is stabilized by DAXX (death domain-associated protein), although, in response to DSBs, the ATM-dependent phosphorylation of DAXX weakens the MDM2-DAXX interaction, which facilitates p53 activation [126]. Together, these mechanisms lead to stabilization and nuclear accumulation of p53, which in turn promotes transcriptional activation of the CDK inhibitor p21. p21 inhibits CDK2-cyclinE activity and causes cell-cycle arrest at the G1/S transition [127–129].

The S phase of the cell cycle is regulated by two checkpoints: one that signals DNA damage (intra-S) and a second that is activated by replication stress (replication checkpoint). In the presence of DSBs, ATM-dependent signaling is also involved in the regulation of the intra-S phase checkpoint through the activation of many downstream kinases. These include CHK1 and CHK2, which phosphorylate CDC25A, to cause inhibition of CDK2 activity and cell-cycle arrest [130]. Another mechanism involved in intra-S checkpoints consists of the direct phosphorylation of CHK2 (T68) by ATM, which can facilitate CHK2 interactions with other proteins, such as BRCA1 and 53BP1 [131] (Figure 2). The ATM-dependent phosphorylation of CHK2 in S phase triggers the subsequent phosphorylation of the phosphatase CDC25A. Once phosphorylated, CDC25A undergoes degradation, which prevents CDC45 from loading onto replication origins, which is required for the initiation of DNA replication [112]. Another pathway depends on ATM, NBS1, BRCA1, and SMC1, which mediate ATM-dependent phosphorylation of the SMC1 and SMC3 subunits of the cohesion complex, and leads to chromosome repair and cell survival [132–135]. Overall, although the exact mechanism leading to activation of intra-S checkpoint signaling remains to be elucidated, ATM/Tel1 and ATR/Mec1 signaling following DNA damage modulate the rate of DNA synthesis and recombinational repair.

The G2/M checkpoint prevents cell entry into mitosis when DNA damage persists. In most vertebrates, this is accomplished by the inhibition of CDK activity, which is regulated by phosphorylation of a conserved tyrosine residue. In contrast, in yeast, this checkpoint acts through inhibition of metaphase to anaphase transition. Rad53 and Chk1 arrest the entry into anaphase, in part through inhibition of Pds1 degradation, while, in a parallel pathway, Rad53 prevents exit from mitosis by the maintenance of high levels of CDK activity [136, 137].

## 5. ATM/Tel1 and Telomere-Length Regulation

**Figure 3:**
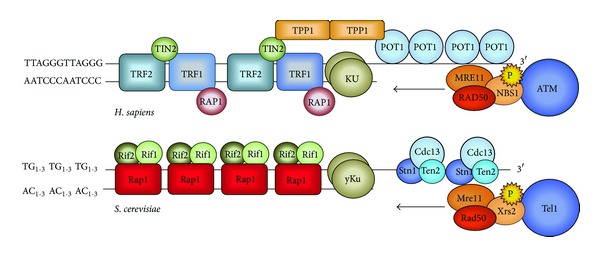
Telomere structure in human and* S. cerevisiae*. Human telomeres consist of kilobases of TTAGGG repeats, ending with a 3′ overhang, G-rich strand. The shelterin complex includes six proteins: TRF1 and TRF2, which bind directly the double-stranded telomeric DNA and are held together by TIN2, RAP1 that interacts with TRF2, POT1 that associates with telomeric ssDNA, and TPP1. These factors mediate the generation of higher-order structure at chromosome ends (T-loop) by invasion of the single-stranded G-overhang into the double-stranded TTAGGG repeats. In Budding yeast, the double-stranded telomeric sequence is bound by Rap1, which regulates telomere length together with Rif1 and Rif2. Cdc13 Ten1 and Stn1 bind to the single strand overhang. In both human and* S. cerevisiae*, the heterodimeric Ku complex (Ku70/80) interacts with the terminal part of the telomere, providing a protective role. The heterotrimeric complex MRX/MRN (MRE11/Mre11, RAD50/Rad50, and NBS1/Xrs2) promotes ATM/Tel1 recruitment, with a central role in telomere capping and length regulation.

Telomeric DNA in most eukaryotes consists of variable numbers of G-rich repetitive elements (TG_1-3_ in* S. cerevisiae* and T_2_AG_3_ in vertebrates), which end with a 3′ single-stranded overhang (G-tail) (Figure 3). The addition of telomeric repeats relies on the activity of the telomerase enzyme [138], a specialized reverse transcriptase that compensates for the erosion that results from the inability of the semiconservative DNA replication machinery to fully replicate chromosome ends [139, 140].

In human, telomerase comprises the catalytic component hTERT, the human telomerase RNA (hTR or hTERC), and dyskerin (DKC1) [141, 142]. Similarly, the yeast telomerase comprises the catalytic subunit Est2, the RNA component TLC1, and two additional proteins Est1 and Est3, which provide essential functions for telomere replication and stability/capping [143–145].

Telomerase recruitment to telomeres appears to be regulated by other proteins that can bind directly or indirectly to telomeric DNA and ensure telomere capping (Figure 3). The capping complex, called shelterin in mammals [146], has a fundamental role in telomere homeostasis, as it provides protection against an incorrect DNA-damage response or inadvertent activation of ATM/Tel1 and ATR/Mec1 signaling [147, 148], as well as allowing telomerase-mediated telomere lengthening. Generally speaking, the capping complex guarantees that only critically short telomeres are subjected to lengthening, whereas average size telomeres are protected from DNA modifying enzymes (telomerase, exonucleases) and do not elicit DDR. Telomere capping proteins in budding yeast comprise the CST complex (Cdc13-Stn1-Ten1), which binds to ssDNA, Ku (Yku70–Yku80), and Rap1-Rif1-Rif2. Similarly, in mammalian cells, the shelterin complex is composed of TIN2, TRF1, TRF2, TPP1, POT1, and RAP1, which provide higher-order DNA structures, the T-loop of which might participate in telomere protection [146, 149, 150].

In mammals and in yeast, ATM/Tel1 deficiency correlates with telomeres shorter than wild-type cells, which reveals a role in telomere-length regulation [19, 21, 22, 151], possibly in directing/limiting telomere lengthening to the shortest telomeres. According to this view, preferential lengthening of the shortest telomeres by telomerase has been shown [152–154]. However, while in* S. cerevisiae* telomerase recruitment to short telomeres appears to be Tel1 dependent, in mammalian cells, ATM is dispensable for the preferential association of telomerase at eroded telomeres [155].

### 5.1. Tel1 in* S. cerevisiae* Telomere-Length Regulation

*TEL1* was originally identified in a screen for genes that affect telomere length in* S. cerevisiae* [21]. In budding yeast,* TEL1* deletion results in dramatic telomere shortening and activation of telomere recombination events [156]. Cells lacking Tel1, as well as* tel1* kinase-dead mutants, have very short, but stable, telomeres, with a length of 50 bp to 100 bp [21]. This suggests that the regulatory role of Tel1 relies on its kinase domain [157, 158]. Also, the second checkpoint kinase, Mec1, appears to have a role in telomere length regulation. Although* mec1* mutants do not show telomere-length variations with respect to wild-type cells, double* tel1*,* mec1 *mutants show progressive telomere attrition and cell senescence reminiscent of telomerase-minus cells [24, 159, 160]. Telomere attrition in the double kinase-deleted cells for* tel1*,* mec1* can be overcome by forcing telomerase loading to telomeres using Est2-Cdc13 fusion, which suggests that Tel1 and Mec1 operate in two different epistasis groups to regulate telomerase recruitment to telomeres [161]. Accordingly, the telomerase activity in mutant cells that lack both Tel1 and Mec1 is indistinguishable from that in wild-type cell [161, 162].

In wild-type cells, Tel1 binding to telomeres appears to be low and limited to the late S-G2 phase of the cell cycle [93]. However, when telomeres are artificially shortened, Tel1 binding increases throughout the cell cycle and remains high for at least two consecutive cycles, which suggests preferential binding of short telomeres [93, 163, 164]. Binding of Tel1 to telomeres requires the MRX complex, and in particular, the interaction with the carboxyl terminus of the Xrs2 subunit of the MRX complex is responsible for MRX recruitment/loading [93]. However, MRX localization is reduced in cells that lack Tel1 [165], which suggests a feedback loop operated by Tel1 on MRX recruitment to telomeres. Of note, disruption of the MRX complex due to* rad50* deletion induces telomere shortening similar to* tel1 or tel1*,* rad50* double mutants, which confirms that Tel1 and MRX work in the same pathway of telomere-length regulation [166].

Live-cell imaging has revealed that yeast telomerase stably associates with a few telomeres only in late S phase of the cell cycle and that, in addition to Tel1 and MRX, this association requires the Cdc13 and Rif1/2 proteins. In particular, it was shown that, in cells that lack Tel1, the clustering of the telomerase RNA component (TLC1) at telomere is disrupted [167]. Additional evidence has shown that Tel1 and the MRX complex preferentially bind short telomeres, which in turn become the substrate for telomerase-mediated telomere lengthening [93, 163, 164, 168]. Therefore, cells that lack Tel1 have short telomeres, due to the reduced frequency of EST1 and EST2 telomerase subunit recruitment and TLC1 RNA clustering at telomeres [167, 169]. Moreover, Tel1 directly phosphorylates Cdc13, which mediates telomerase recruitment through interaction with the telomeric G-tails and the Est1 subunits of telomerase [170].

The preferential targeting of Tel1 and MRX to short telomeres depends on the Rap1-Rif2 complex (Figure 3). According to the counting model, as telomeres get shorter, the number of Rap1-Rif2 molecules decreases [171, 172] and elicits the signal for MRX and Tel1 and ultimately telomerase recruitment. In support of this model, it has been reported that the preferential binding of Tel1 to short telomeres is lost when Rif2 is mutated and that Rif2 directly interacts with MRX [165, 168, 173]. Nevertheless, by artificially altering the sequence of the yeast telomeres in such a way that Rap1 binding is lost, though slightly shorter, the telomeres are stably maintained in dividing cells, and* TEL1* deletion affects their length similarly to wild-type cells [174–176]. This suggests that there is a Rap1-independent mechanism of telomere regulation [177]. Interestingly, in these strains, the roles of Tel1 in G-tail processing and preferential binding to short telomeres are maintained [166, 178].

Mainly based on the observations that Tel1 phosphorylates the telomerase recruitment domain of Cdc13 [170] and associates to telomeres in a length-dependent manner, the most commonly accepted model of Tel1 activity proposes that Tel1 preferentially binds short telomeres and promotes the recruitment of the telomerase enzyme. Thus at a cellular level, Tel1 restricts lengthening to the shortest telomeres. However, some data are in contrast with this interpretation. The preferential elongation of short telomeres still occurs at native telomeres in tel1 mutants [25]; additionally, cell senescence in telomerase-minus cells is attenuated in the absence of Tel1. These findings suggest an alternative model by which the reduced telomere shortening in these tel1 mutants, the telomerase-minus cells, is due to reduced telomere resection, which in turn delays the onset of critically short telomeres leading to senescence [26, 179]. Therefore it remains uncertain if Tel1 directly phosphorylates specific targets at telomeres, to promote telomerase recruitment, or if it indirectly stimulates the G-tail lengthening that provides a favorable substrate for telomerase association [26].

### 5.2. ATM in Mammalian Telomere-Length Regulation

In budding yeast, Tel1 is crucial for telomerase recruitment to short telomeres, while ATM appears to be dispensable for this function in human [155]. Nevertheless, mammalian telomerase maintains an apparent selective preference for critically short telomeres [153, 180, 181]. A lot of evidence has strongly suggested that ATM participates in telomere maintenance, which includes the finding that primary and immortalized AT cells show accelerated telomere shortening, chromosome fusions, premature aging, and a senescent phenotype [17, 19, 182]. Double deficiency for ATM and telomerase in mice (ATM^−/−^  TER^−/−^) induces more rapid telomere erosion and genome instability [183]. Moreover, the simultaneous knock-out of ATM and TER leads to a higher rate of germ-cell death and chromosomal fusions, relative to mice with a single gene mutation. This appears to suggest that ATM deficiency results in more prominent telomeric dysfunction [184].

It has been shown that ATM influences the fraction of telomeres that are attached to the nuclear matrix [182], as shown by the finding that a higher percentage of telomeric DNA (80%) is anchored to the nuclear matrix in ATM-deficient cells, with respect to the wild-type cells (50%) [182]. These data might correlate to the higher rate of telomere erosion and to telomere fusions observed in AT cells.

Overall, ATM-deficient cells appear to have some dysfunctions that are typical of uncapped telomeres, which suggests that ATM acts in concert with the shelterin complex (Figure 3), to guarantee full telomere protection. Accordingly, it has been shown that telomere fusions result from ATM-dependent activation of the DDR, in mouse embryonic fibroblasts conditionally deleted for the shelterin component TRF2. This outcome, together with other studies carried out in human cell lines, suggests that ATM activity at telomeres is repressed by TRF2 [185, 186]. Indeed, the overexpression of TRF2 causes inhibition of the ATM-mediated response to DNA damages after exposure to ionizing radiation and abrogates cell-cycle arrest by the reduction of p53 activation. ATM inhibition mediated by TRF2 requires direct interactions between the two proteins (the region of ATM containing the S1981 site), which blocks ATM activation [187]. As TRF2 is abundant at telomeres, the inhibition of ATM might prevent recognition of telomeres as a site of DNA damage without affecting the surveillance of internal chromosome breakage [187].

ATM interacts also with TRF1, another element of the shelterin complex, through a domain that is different from that used to contact TRF2 [187–189]. How ATM is involved in telomere-length regulation is suggested by experiments performed in human fibroblastoma cells and in primary fibroblasts expressing telomerase. In these cells, ATM inhibition results in reduction of phosphorylated TRF1 and a consequent increase in TRF1 association to telomeres, which leads to telomere shortening. Moreover, the increased association of TRF1 at telomeres depends on the MRN complex, as it is abrogated in cells lacking MRE11 or NBS1. These data suggest a model by which MRN is required to promote ATM-dependent TRF1 phosphorylation and its subsequent release from telomeres, thus promoting telomerase recruitment [190]. According to this view, MRN deficiency, induced by RNA interference, caused G-tail shortening in telomerase-positive cells but not in telomerase-negative cells. This suggests that the resection activity of the MRN complex is somehow connected to telomerase recruitment and/or activity [191]. The most reliable explanation is that MRN and ATM cooperate to regulate telomere resection and capping, so that optimal G-tails for telomerase recruitment are produced.

This is confirmed by specific diseases that are linked to single mutations in the genes that compose the MRX complex, the symptoms of which resemble those of AT patients. Mutations in the* NBS1* gene result in a rare autosomal recessive disorder called Nijmegen breakage syndrome (NBS). In the absence of NBS1, phosphorylation of ATM is incomplete, and this speeds up telomere shortening, defective activation of the apoptotic pathway, and accumulation of chromosomal instability [192, 193]. Additionally, mutations affecting one of the other two members of the MRN complex, MRE11 and RAD50, have been linked to the onset of ataxia-telangiectasia-like disorders [194].

Thus, the emerging picture is that ATM has a complex role also at mammalian telomeres, through interactions with the shelterin proteins TRF1 and TRF2 and with the MRN complex, to ensure telomere protection and length regulation. In particular, telomerase recruitment and the telomerase-mediated telomere elongation pathway resemble the telomere regulation process that is controlled by Tel1 kinase in budding yeast, which indicates the presence of an evolutionary conserved mechanism [190].

## 6. ATM Deficiency in Cancer Predisposition

Even before the cloning of the ATM gene, it was evident that AT patients were affected by a high incidence of cancer, in particular thymus, breast cancer, lymphoma, and leukaemia [195]. The higher incidence in the development of leukaemia and lymphoma, described in AT patients, has been related to the decreased ability of AT cells to correctly control the DSBs that physiologically occurs during the maturation of the immune system [196]. Indeed, the DDR represents a central event of the V(D)J recombination. This is a programmed DNA rearrangement process that occurs during the early development of lymphocytes and that allows the assembly of highly diversified antigen receptors essential to functional lymphocytes. Therefore, ATM deficiency affects the V(D)J recombination-induced DSBs preventing the production of antigen receptors, compromising T- and B-cell developments and causing severe immune deficiencies. This is also confirmed by experiment in Atm^−/−^ mice that develop lymphoma and leukaemia within the first three months of life and die of malignant thymic lymphoma by 4-5 months of age [183, 197].

In 1987, Swift et al. reported that the incidence of breast cancer was significantly higher in female relatives of patients affected by an autosomal recessive condition of AT [198]. However, many studies in the following years failed to convincingly associate ATM with breast cancer [199–201]. Only recently did an extensive study of gene mutational screening in patients affected by non-BRCA1/BRCA2 familial breast cancer clearly categorize ATM as a breast cancer gene [202].

By now, many ATM mutations have been reported to increase cancer predisposition, including truncation and missense mutations [201, 203–206]. This phenomenon is clearly related to the multiple roles of ATM in DDR, including the control and signaling of DNA lesions, which results from different stimuli, such as endogenous oxidative DNA damage, mutagens, breaks occurring at meiosis, and gene rearrangements [207]. ATM provides strong tumor suppressive effects by activation of cell-cycle arrest and apoptosis in cancer cells via the interaction with p53 [208]. Phosphorylation of deleted in breast cancer 1 (DBC1) by ATM inhibits SIRT1 deacetylase (one of seven mammalian orthologs of the yeast protein silent information regulator 2, Sir2), a regulator of p53. Conversely, depletion of DBC1 increases SIRT1 activity, which in turn promotes deacetylation of p53, thus providing protection from apoptosis [209] (Figure 2).

It has been reported that ATM can act as a tumor suppressor in liver cancer, by directly phosphorylating Tax1 binding protein 2 (TAX1BP2), a cyclin-dependent kinase 2-regulated tumor suppressor. TAX1BP2 phosphorylation stabilizes this protein and activates the p38 MAPK/p53/p21 pathway [210]. Cancer predisposition among AT carriers has revealed that the high rate of malignancy, in particular in breast cancer, is frequently associated with ATM heterozygosity [211, 212]. It has been estimated that heterozygotes, with ATM mutations that are present in as many as 1% of the total AT population are exposed to an associated risk of the development of breast cancer that is three-to-five-fold greater than the rest of the people [213, 214]. ATM heterozygous mutations have been identified by genome-wide sequencing analysis in the germline of nearly 170 patients with history of pancreatic cancer posing ATM as a new potential target gene for predisposition of pancreatic ductal adenocarcinoma [215]. From the Catalogue Of Somatic Mutations In Cancer (COSMIC) it emerged that, from 8901 samples of all cancer types catalogued, with some tissue-dependent variations, 5% have ATM mutations and this data might underestimate the real impact of ATM aberrations in cancer [216]. A detailed analysis of the data from The Cancer Genome Atlas (TCGA) consortium set for glioblastoma multiforme (GBM), the most common and lethal primary central nervous system tumor in adults, shows that 3.2% of tumours have somatic mutations in ATR, ATM, or CHK1 [217]. The tumour sequencing project (TSP), a large-scale exon-directed sequencing experiment to classify recurring somatic mutations in lung adenocarcinoma, found that 7% of 188 lung adenocarcinoma patients analysed harboured mutations in ATM [218]. The TSP identified 10 missense mutations, 2 frameshift deletions, a splice site mutation, and a nonsense mutation, consistent with loss of function.

The large body of literature produced over the years has reported that ATM variants can have different and frequently opposing effects in cancer predisposition, which causes a multitude of phenotypes.

When Renwick et al. categorized patients with family histories of breast cancer, it emerged that known AT-causing variants were associated with only a moderate increase in breast cancer predisposition [202]. However, in this study, the distinction between the effects of different types of ATM mutations was not considered. In 1999 Gatti et al. hypothesized that AT heterozygous carriers, which have one truncated version of the protein, behave differently from those with a missense mutation that might act as a dominant negative, which confers particularly high risk of breast cancer [219]. Hence, AT carriers with truncating mutations fail to produce any ATM protein, and carriers have almost the wild-type phenotype, relying only on the activity of the functional ATM allele. On the contrary, some missense mutations produce abnormal proteins that are present at normal levels inside cells. The molecules with missense mutation compete with the normal ones for the target substrates, thus acting as dominant negatives. This condition can explain why, in many cases, AT heterozygous with ATM in missense mutations is associated with a high risk of cancer incidence with respect to heterozygous with a truncated version of ATM [220].

The impact of ATM missense mutations also came from the study of patients affected by sporadic human tumors. These somatic mutations were largely missense, and in many cases, ATM behaved like a tumor suppressor [221]. Interestingly, according to the tumor suppressor activity, ATM was downregulated in 55% of 119 patients with breast cancer, compared with adjacent normal breast tissues [222]. It has been reported that the microRNA miR18a can impair DDR through downregulation of ATM expression [223]. Additionally, aberrant overexpression of miR421 influences ATM posttranscriptional downregulation [224, 225] and this is associated with poor prognosis in sporadic breast cancer [226]. Reduced ATM mRNA abundance significantly correlates with aberrant methylation of the ATM promoter, which suggests that epigenetic silencing of ATM expression can occur in breast cancer. However, a precise correlation between ATM methylation and its expression is still debated [227, 228]. Low expression of ATM observed in breast cancer tissue was frequently related to the accumulation of high rates of DNA mutations and to tumor progression; however, ATM expression is a complex process, and breast cancer onset can be influenced by several mechanisms. Indeed, other data do not support the suppressor role of ATM as no defective expression of ATM has been observed in sporadic breast cancers [229]. Paradoxically, upregulation of ATM in prostate and pancreatic cancer cells has been frequently reported, linking this condition to those cells that have somehow escaped cell-cycle arrest and apoptosis [230, 231]. Increased ATM expression can also be associated with a more efficient DNA damage response, as the oncogenic activation can cause replication stress. This condition also correlates with an increment in chemoresistance and radioresistance that promote the survival and invasive behavior of metastatic cells [232].

Overall, these observations reveal the complex architecture that characterizes the activity of ATM in the DNA-damage response, the maintenance of genetic stability, and cell-cycle regulation, and how this multifunctional activity correlates with genetic predisposition or sporadic onset of cancer.

## Figures and Tables

**Table 1:** Components of the *Saccharomyces cerevisiae* DNA damage response pathway and their orthologs in *Homo sapiens*.

*H. sapiens *	*S. cerevisiae *	Description
ATM	Tel1	Protein kinase- (PIKK-) DNA damage response and telomere length regulation
ATR	Mec1	Protein kinase- (PIKK-) DNA damage response and telomere length regulation
MRE11-RAD50-NBS1	Mre11-Rad50-Xrs2	DSB sensing, nuclease
CHK2	Rad53	DNA damage response protein kinase; checkpoint effector
CHK1	Chk1	Protein kinase; checkpoint effector; mediates cell-cycle arrest
CtIP	Sae2	Endonuclease
EXO1	Exo1	5′-3′ Exonuclease
BLM	Sgs1	DNA helicase
DNA2	Dna2	ATP-dependent nuclease and helicase
RAD9-RAD1-HUS1	Ddc1-Rad17-Mec3	Checkpoint clamp (9-1-1 complex)
53BP1; BRCA1; MDC1	Rad9	DNA damage-dependent checkpoint protein
